# Nutritional support in the tertiary care of patients affected by chronic renal insufficiency: report of a step-wise, personalized, pragmatic approach

**DOI:** 10.1186/s12882-016-0342-3

**Published:** 2016-09-06

**Authors:** Adamasco Cupisti, Claudia D’Alessandro, Biagio Di Iorio, Anna Bottai, Claudia Zullo, Domenico Giannese, Massimiliano Barsotti, Maria Francesca Egidi

**Affiliations:** 1 Department of Clinical and Experimental Medicine, University of Pisa, Via Roma 67, 56126 Pisa, Italy; 2 U.O.C. di Nefrologia, PO “A. Landolfi”, PO di Solofra (AV), Solofra, Italy

## Abstract

**Background:**

Dietary treatment is helpful in CKD patients, but nutritional interventions are scarcely implemented. The main concern of the renal diets is its feasibility with regards to daily clinical practice especially in the elderly and co-morbid patients. This study aimed to evaluate the effects of a pragmatic, step-wise, personalized nutritional support in the management of CKD patients on tertiary care.

**Methods:**

This is a case-control study. It included 823 prevalent out-patients affected by CKD stage 3b to 5 not-in-dialysis, followed by tertiary care in nephrology clinics; 305 patients (190 males, aged 70 ± 12 years) received nutritional support (nutritional treatment Group, NTG); 518 patients (281 males, aged 73 ± 13 years) who did not receive any dietary therapy, formed the control group (CG). In the NTG patients the dietary interventions were assigned in order to prevent or correct abnormalities and to maintain a good nutritional status. They included manipulation of sodium, phosphate, energy and protein dietary intakes while paying special attention to each patient’s dietary habits.

**Results:**

Phosphate and BUN levels were lower in the NTG than in the CG, especially in stage 4 and 5. The prevalence of hyperphosphatemia was lower in the NTG than in CG in stage 5 (13.3 % vs 53.3 %, *p* < 001, respectively), in stage 4 (4.1 % vs 18.3 % vs, *p* < 0.001) and stage 3b (2.8 % vs 9.5 % *p* < 0.05). Serum albumin was higher in NTG than in CG especially in stage 5 . The use of calcium-free intestinal phosphate binders was significantly lower in NTG than in CG (11 % vs 19 % *p* < 0.01), as well as that of Erythropoiesis stimulating agents (11 % vs 19 %, *p* < 0.01), and active Vitamin D preparations (13 % vs 21 %, *p* < 0.01).

**Conclusions:**

This case-control study shows the usefulness of a nutritional support in addition to the pharmacological good practice in CKD patients on tertiary care. Lower phosphate and BUN levels are obtained together with maintenance of serum albumin levels. In addition, a lower need of erythropoiesis stimulating agents, phosphate binders and active Vitamin D preparations was detected in NTG. This study suggests that a nutritional support may be useful in the management of the world-wide growing CKD burden.

## Background

Nutritional therapy is a part of medical treatment of pre-dialysis chronic kidney disease (CKD), and it is historically related to manipulation of dietary protein intake [1, 2]. The most frequently applied schemes include the “low-protein” (0.6–0.7 g/Kg/day) diet or the “very low protein” (0.4–0.3 g/Kg/day) diet supplemented with essential amino acids and ketoacids [3]. Although relevant, protein restriction is only one aspect of the dietary management of CKD patients. Additional aspects include modifications in sodium, phosphorus and energy intake, as well as in the source (animal or plant derived) of protein and lipids [4]. Information about processed foods and home-based food preparations are additional modifiable factors useful to modulate phosphate and sodium effective load. As a whole, the aim of the nutritional support is to prevent or correct signs and symptoms of renal failure, potentiate drug therapy, and to postpone the initiation of dialysis while maintaining nutritional status [5, 6]. Despite evidences that dietary treatment is helpful in CKD patients, nutritional interventions are scarcely implemented in renal clinics worldwide. Currently, the main concern of the renal diets is its feasibility with regards to daily clinical practice especially in the elderly and co-morbid patients [6]. This point is particularly timely due to changes of the features of CKD patients followed in the renal clinics. The prevalence of diabetes and cardiovascular co-morbid conditions is increasing as well as the age of the patients. A recent epidemiological study in Italian renal clinics showed that the mean age of pre-dialysis CKD patients was 71 years [7]. It is noticeable that in the MDRD study (1992–1993), the largest study on the effect of protein restriction in CKD, the patients’ average age was 52–53 years old and more importantly diabetics and patients older than 70 years were excluded [8]. So it is quite difficult to transpose those data on the actual CKD population. The increasing age of the CKD patients is associated with lower spontaneous food intake which is also a function of the severity of renal insufficiency [9]. Therefore a restrictive dietary approach, mainly guided by the level of residual renal function [3] may be not suitable to address the clinical needs of the current CKD population and may be at risk of protein-energy wasting.

Herewith we report a practical approach that we experienced in our renal nutrition clinic. It was based on the assessment of the patient’s habitual energy and nutrients intakes, in order to define the dietary interventions to correct metabolic or nutritional abnormalities. Attention was payed to avoid dramatic changes in patient’s eating behavior, to allow for greater chances of concordance, adherence and quality of life. Moreover, nutritional approach for CKD patients should include psychosocial factors and behavioral aspects, such as participant knowledge, attitude, support, satisfaction, self-monitoring and self-perception of success. The rating of satisfaction of the dietary pattern affects patients adherence to the dietary prescriptions: a dietary intervention that starts from medical recommendations, takes into account patients habits, needs and lifestyle has more chances to guaranty a good adherence to the dietary treatment in the long-term [10].

In this study we evaluated the effects of this pragmatic, patient centered, step-wise nutritional support in the management of CKD patients on tertiary care.

## Methods

This is a case-control study. It included 823 prevalent out-patients affected by CKD stage 3b to 5 not-in-dialysis followed by tertiary care in nephrology clinics in the period 2012–2015. Exclusion criteria were eGFR >45 ml/min *1,73 m^2^, acute kidney injury, cancer, kidney transplanted patients, or patients on acute illness or on immunosuppressive therapy.

Three hundred and five patients (190 males and 115 females, aged 70 ± 12 years) who received nutritional counseling and were on dietary treatment in our CKD clinic, for 6 months at least, formed the Nutritional Treatment Group (NTG). The control group (CG) included 518 patients (281 males and 237 females, aged 73 ± 13 years) who did not receive any nutritional support or dietary therapy: they were recruited from the Italian Nefrodata Study cohort [7]. The Italian Nefrodata is a multicentric, prospective, observational study conducted in Italy. It included 1263 patients with CKD stage 3–5 on a tertiary care setting who were given good-practice pharmacological therapy. From this cohort we extracted the baseline data of the prevalent patients who were not given any nutritional support. The prevalence of diabetes was similar in the NTG (35.7 %) and in the CG (34.4 %) as well as the prevalence of cardiovascular co-morbidities (30.9 and 27.8 %, respectively).

Clinical and biochemical data were obtained from the medical files. Biochemistry was performed using routine lab methods.

The prevalent use of ACE-inhibitors or Angiotensin II receptor blockers, furosemide, statins, allopurinol, calcium carbonate and non-calcium containing phosphate binders, active Vitamin D preparations and Erythropoiesis stimulating agents (ESAs) was recorded in both groups.

A subgroup of 109 NTG patients was given the Dietary Satisfaction Questionnaire, a 30 item form developed by the MDRD Nutrition Coordinating Center at the University of Pittsburg, on the basis of a questionnaire of the Case Western Reserve University [10]. It was completed by the patients out of the dietician office. The purpose of the Dietary Satisfaction Questionnaire was to assess the patients’ feelings about their eating patterns, by questions addressing quantity and quality of foods, difficulties in meal preparation and planning and attitude to changes in dietary habits [10]. The answers to the first item of the Dietary Satisfaction Questionnaire was designed to assess the overall satisfaction with diet: “Rate your overall satisfaction with the way you are currently eating” In this paper we report the prevalence of the patients’ responses that were taken from 1 (dislike extremely) to 5 (like very much). The responses of dislike extremely (1) and dislike (2) were combined and referred as “dislike”, whereas liked (4) and liked very much (5) were combined and reported as “like”; the score 3 was considered as “neutral”. The responses of the other items were well in keeping with the first with regards to motivation in following the diet, how many times the diet is observed, the availability of finding food necessary for the diet and the organization of the meals [10].

The NTG patients received nutritional support consisting of a “step-wise “, personalized approach, by a registered renal dietician [11]. Following the clinical evaluation by the nephrologist, the renal dietitian assessed dietary habits by a 3-day dietary recall, and performed an intervention tailored to the needs and clinical features of the patient. Currently, dietary prescriptions were assigned not merely as a function of residual renal function, but towards the correction of abnormalities (if any) and to the maintenance of a good nutritional status.

As a preliminary objective, the patient was requested to follow healthy dietary habits (HDH); in particular suggestions were given to reduce salt intake and to limit excess animal protein and phosphate intake.

The first step was to plan a “normal” diet (ND) that is a diet that equals WHO recommendation for the general population, namely a dietary protein intake of 0.8 g/Kg/d and salt intake of 5–6 g per day [12, 13]. Grains, legumes consumption was encouraged as well as vegetables and fruits with some precautions in the case of hyperkalemia.

When the “normalization” of protein intake was not enough to maintain a good metabolic control (i.e. BUN < 50 mg/dl, serum phosphate < 4.0 mg/dl, bicarbonate > 23 mmol/l), dietary protein restriction was proposed as the second step. A low protein diet (LPD) supplying 0.6 g of proteins per Kg of body weight is enough to cover protein needs provided that there is an adequate amount of foods rich in high biological value protein (meat, fish and white egg in particular), and an adequate energy intake. For this reason the use of protein-free products was generally recommended as they represent a source of energy without wasting products [14].

A low protein (0.7 g/Kg/day) vegan diet (VD) was an alternative to the animal-based LPD [15–18]. In the vegan diets the consumption of mixtures of grains and beans is mandatory to guarantee a an adequate essential amino acids intake [15].

When more severe restrictions were needed to correct the metabolic abnormalities, a very low protein (0.3–0.4 g/Kg/day) diet (VLPD) was prescribed as the third step. The VLPD needs supplementation of a mixture of essential amino acids (EAA) and ketoacids (KA) and an energy intake that equals or even surpasses the energy requirement [19]. At each level of dietary protein intake, phosphate intake was as low as possible, taking care to avoid processed food and those containing preservatives, favoring plant-origin food and using boiling as cooking method [20].

Protein-free products and EAA and KA represent useful tools in the dietary management of CKD patients. Protein-free products are useful for the safe and successful implementation of an animal-based LPD or a very low protein diet for CKD patients. They represent a source of energy from carbohydrates free from nitrogen, and with a low to negligible content of potassium, sodium and phosphorus. They are generally used for the implementation of low protein diets but they can be used also as an additional source of energy when needed [14].

The mixture of EAA and KA have generally been used to supplement VLPD in patients with advanced CKD but they can be also given when spontaneous protein intake results insufficient. For example it is a quite common finding that elderly people have monotonous dietary habits that result in a spontaneous reduction of protein and energy intake. In these cases, the priority is to let them eat enough and to avoid restrictions while the supplementation with EAA and KA is used to achieve an adequate nitrogen intake to prevent/correct protein-energy wasting.

As a general rule, patient’s dietary habits were modified as little as possible. In the case of low-nutrient intake, which frequently occurs in elderly patients, supplements or protein-free products can be added as source of energy and/or EAA and KA can be prescribed above patient’s habitual diet.

In the daily clinical practice, the role of the patient is pivotal to obtain the success and safety that is expected by these complex dietary approaches. So a proper counseling is required for patient’s concordance and adherence. Practical advice consists of defining the amount of foods rich in animal proteins (using domestic measures), reducing foods rich in salt (such as processed meats) and dairy products, that are also rich in phosphorus. To limit overly restrictive prescriptions, we address the frequency of consumption. For example in those patients who like cheeses (that have a high sodium and phosphate content), we suggested to eat cheeses once a week or every 10 days instead of prohibiting them. This was a well accepted approach and contributed to a better adherence to nutritional therapy.

Boiling was suggested as the most useful method of cooking to reduce the mineral content of foods [21]. Practical advice and recipes were given to improve taste and appearance of foods. The use of olive oil, source of healthy fats (to be limited in the case of overweight/obesity), herbs and spices helps in achieving this goal. Last but not least, strong recommendations were given to avoid processed foods or foods and beverages with phosphate-containing preservatives [22].

Patients following a VLPD were supplemented with EAA and KA (1 tablet every 5 Kg of body weight). Sodium bicarbonate or cholecalciferol where given when metabolic acidosis or hypovitaminosis D were detected. Low dose calcium carbonate (1 g/day) was supplemented in the cases of low-phosphate diet because it is poor in calcium, as well.

### Statistical analysis

Descriptive analysis is reported as Mean ± SD and percentage. Statistical analysis was performed by student’s *t* test for unpaired data or chi-square test. Differences were considered as statistically significant when *p* < 0.05.

## Results

Within each CKD stage group, eGFR were very similar between the NTG and the CG patients. The prevalence of diabetes or cardiovascular co-morbidities (myocardial infarction, cerebrovascular disease or obstructive lower-limb vascular disease) by CKD stages is reported in Table 1. Arterial blood pressure control was satisfactory and similar in both groups, at the same CKD stage (Table 1). At the same eGFR level, BUN was lower in the NTG than in the CG, especially in stage 4 and 5 (Table 1).

**Table 1:** Age, Body Mass Index, Arterial blood pressure values and biochemistry in Nutritional Therapy Group (NTG) and Control Group (CG) patients by CKD stages

	Stage					
	CKD 3b		CKD 4		CKD 5	
	*NTG* *n = 148*	*CG* *n = 262*	*NTG* *n = 126*	*CG* *n = 204*	*NTG* *n = 31*	*CG* *n = 52*
Age, yrs	70 ± 13	73 ± 12	71 ± 11**	74 ± 13	69 ± 17	69 ± 15
Diabetes, %	34.0	39.6	41.3	31.3	22.5	19.2
CV Morbidity, %	33.1	31.1	31.0	25.9	22.5	19.2
BMI, kg/m^2^	27.9 ± 4.0	28.3 ± 4.7	27.6 ± 5.3	27.7 ± 5.7	26.9 ± 6.5	26.1 ± 4.1
SBP, mmHg	138 ± 18**	131 ± 17	135 ± 16	133 ± 20	136 ± 29	137 ± 20
DBP, mmHg	79 ± 10*	74 ± 10	75 ± 11***	73 ± 10	78 ± 14	77 ± 8
PP, mmHg	59 ± 17	58 ± 15	60 ± 15	61 ± 19	58 ± 20	61 ± 18
eGFR, ml/min*1.73 m^2^	38.2 ± 5.1	36.8 ± 4.1	23.4 ± 4.1	23.3 ± 4.1	12.0 ± 2.6	12.0 ± 2.1
BUN, mg/dl	33.8 ± 9.5	36.7 ± 11.4	41.9 ± 15.2*	49.5 ± 17.1	49.0 ± 24.3*	72.3 ± 16.2
Calcium, mg/dl	9.4 ± 0.5	9.3 ± 0.9	9.4 ± 0.5	9.3 ± 0.7	9.3 ± 1.5	9.1 ± 0.9
Phosphate, mg/dl	3.3 ± 0.6*	3.6 ± 0.7	3.6 ± 0.5**	3.9 ± 0.9	3.9 ± 1.2*	4.7 ± 1.0
PTH, pg/ml	108 ± 65	116 ± 103	160 ± 100	150 ± 112	232 ± 138	154 ± 140
Sodium, mEq/l	140 ± 2	141 ± 3	140 ± 2	140 ± 4	140 ± 2	140 ± 3
Potassium, mEq/l	4.7 ± 0.4	4.7 ± 0.6	4.7 ± 0.6	4.8 ± 0.6	4.6 ± 0.4	4.9 ± 0.8
Haemoglobin, g/dl	13.2 ± 1.8	12.7 ± 1.7	12.2 ± 1.4***	11.8 ± 1.5	11.4 ± 2.0	11.5 ± 1.4
Haematocrit, %	40.3 ± 5.2***	38.5 ± 4.9	37.3 ± 4.2***	36.2 ± 4.8	35.3 ± 6.3	35.7 ± 4.8
Albumin, g/dl	4.1 ± 0.4***	3.9 ± 0.4	4.1 ± 0.4*	3.8 ± 0.4	4.1 ± 0.8**	3.7 ± 0.6
Total cholesterol, mg/dl	178 ± 36	178 ± 42	183 ± 40	177 ± 43	175 ± 49	165 ± 42
LDL cholesterol, mg/dl	100 ± 32	106 ± 34	102 ± 32	99 ± 34	98 ± 32	88 ± 29
HDL cholesterol, mg/dl	51 ± 17	52 ± 29	53 ± 23	49 ± 15	48 ± 17	44 ± 16
Triglycerides, mg/dl	141 ± 68	145 ± 77	151 ± 87	148 ± 76	144 ± 76	133 ± 75
Urate, mg/dl	6.3 ± 1.5	6.1 ± 1.8	6.7 ± 1.9*	5.8 ± 1.9	6.3 ± 3.0	6.0 ± 3.0

Abbreviations: *BMI* (body mass index), *BUN* (blood urea nitrogen), *DBP* (diastolic blood pressure), *PP* (pulse pressure), *SBP* (systolic blood pressure)

*: *p* < .001; **: *p* < .01; ***: *p* < .05 vs CG

In the NTG, phosphatemia was lower than in CG at all the stages of the disease (Table 1): the prevalence of hyperphosphatemia (as defined as sP > 4.5 mg/dl) was lower in the NTG than in the CG in stage 5 (53.3 % vs 13.3 %, *p* < 0.01, respectively), in stage 4 (18.3 % vs 4.1 %, *p* < 0.001) and stage 3b (9.5 % vs 2.8 %, *p* < 0.05).

Serum albumin was lower in the CG than in the NTG especially in stage 5 (Table 1).

In the NTG stage 4 and 5 serum bicarbonate was well controlled (24.7 ± 3.2 and 24.4 ± 2.3 mM, respectively); unfortunately too many missing data in the CG prevented a statistical comparison. Additional data regarding hemoglobin, serum lipids, urate and BMI are reported in Table 1, and they were roughly similar in NTG and CG.

**Fig. 1:**
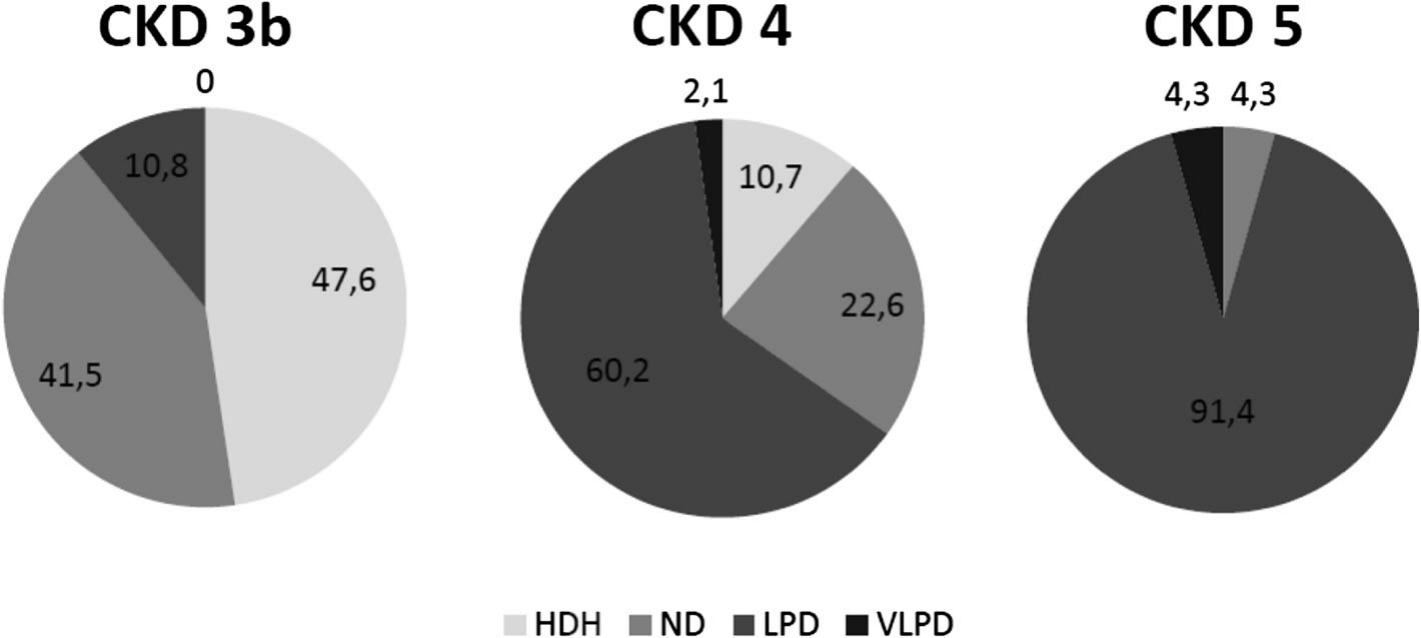
The types of nutritional treatments in the patients of Nutritional Therapy Group, by CKD stages HDH (*healthy dietary habits*), ND (*normal diet*), LPD (*low protein diet*), VLPD (*very low protein diet*)

The nutritional treatments by CKD stages are shown in Fig. 1. As expected, the implementation of a LPD increased from stage 3b (10.2 %) to stage 4 (60.2 %) and stage 5 (91.4 %). In stage 3b a HDH and a ND are largely prevalent. The VLPD was given in a few number of patients, and in selected cases. Meanwhile, a number of patients received EAA and KA supplementation on the top of a LDP, when needed.

**Fig. 2:**
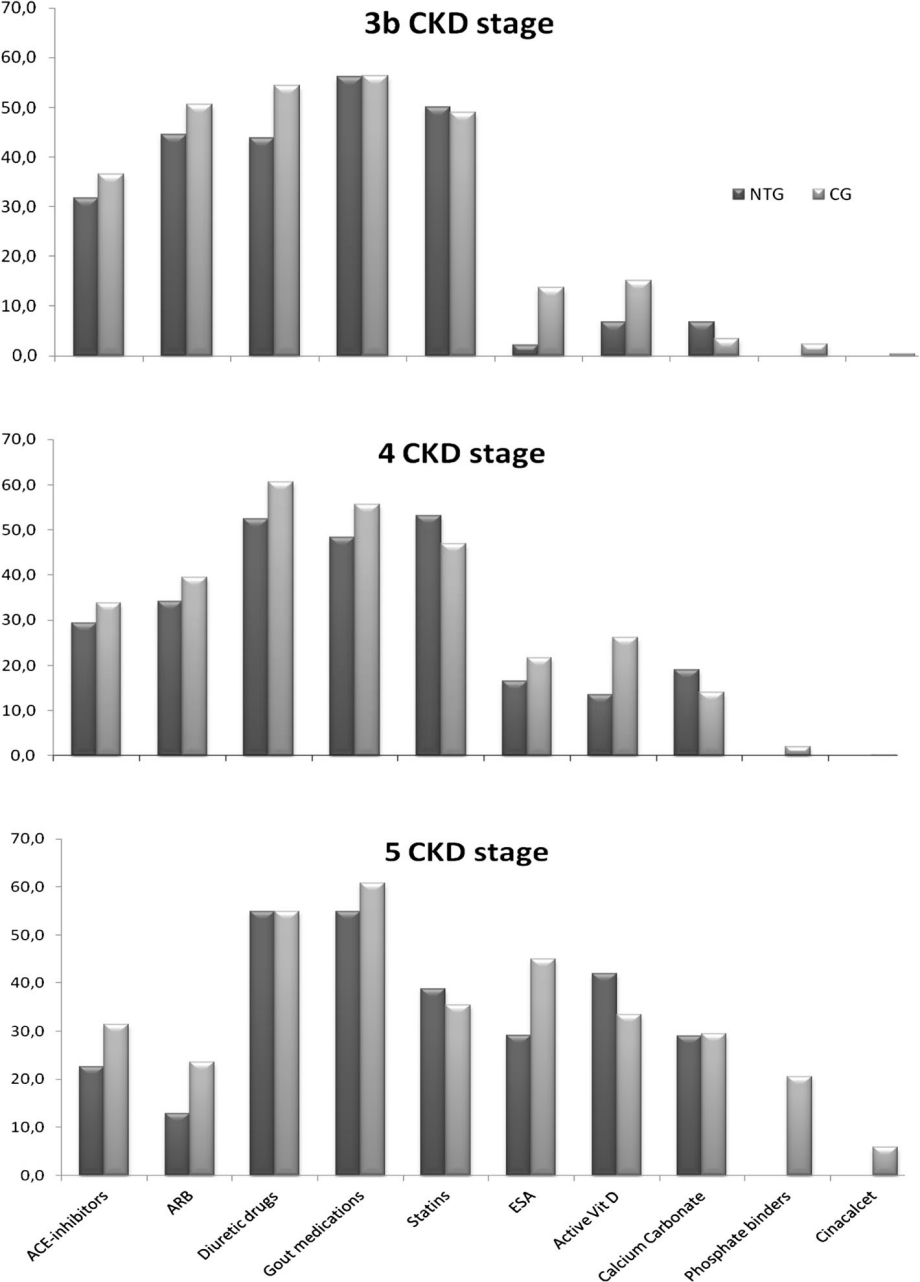
Prevalence of CKD-related pharmacological therapies in the NTG (*dark columns*) and in the CG (*grey columns*) by CKD stages

The prevalence of CKD-related pharmacological treatments by CKD stages in the NTG and in the CG is reported in Fig. 2. As a whole, the prevalence of ACE-inhibitors or Angiotensin II receptor blockers was similar as well as that of statins and allopurinol. Furosemide therapy was less prevalent in the NTG than in the CG (48.5 % vs 56 %, *p* < 0.05). The use of calcium-free phosphate binders was significantly lower in the NTG than in the CG (19 % vs 11 %, *p* < 0.01). Similarly, the prevalence of Erythropoiesis stimulating agents (ESA) therapy was significantly lower in the NTG than in the CG (11 % vs 19 %, *p* < 0.01), as well as that of active Vitamin D preparations (13 % vs 21 %, *p* < 0.01).

**Fig. 3:**
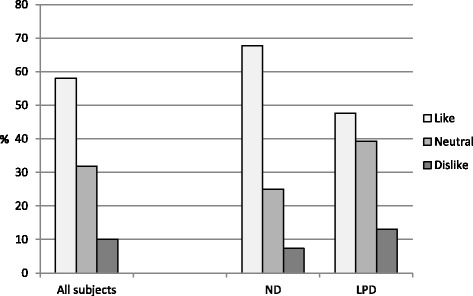
Prevalence of the responses to the question “Rate your overall satisfaction with the way you are currently eating”. The answers “dislike extremely” (*score 1*) and “dislike” (*score 2*) were combined and referred to as “Dislike”; the answers “Like very much” (*score 5*) and “like” (*score 4*) were combined and referred to as “Like”; the rating 3 was considered as “Neutral”. Results are reported for all the 109 subject, and separately for low protein diet (*LPD*) and for normal diet (*ND*) patients

The results of the Dietary Satisfaction Questionnaire showed that the majority of the patients were satisfied with their diet (Fig. 3). The rating of LPD was similar to that of ND (3.3 ± 1.0 vs 3.6 ± 0.89) patients. The prevalence of “dislike” was reported by 7.3 % of ND and by 13.1 % of LPD patients (Fig. 3). As expected, LPD patients had lower eGFR than ND patients (21.9 ± 8.3 vs 37.0 ± 10.5 ml/min*1.73 m^2^, *p* < 0.001).

## Discussion

The results of the present investigation shows that a nutritional support gives additional favorable effect on the metabolic and clinical management of patients affected by CKD 3b-5 and followed in tertiary care centers [5]. It is the major reason why evidence exists that dietary treatment can help to postpone the initiation of dialysis [6].

Both the NTG and the CG patients were followed in tertiary care clinics so that good practice pharmacological therapies were guaranteed. Accordingly, blood pressure control, level of PTH and hemoglobin, urate and lipids were satisfactory and similar in both groups. Meanwhile, parameters potentially affected by nutritional intervention were significantly different. At the same eGFR, BUN was lower in the NTG than in the CG, especially in stage 4 and 5 where LPDs were largely used. Similarly, serum phosphate levels were lower in the NTG than in the CG, as well as the prevalence of hyperphosphatemia. This occurred despite that fact that the use of phosphate binders was more prevalent in the CG patients. It is noteworthy that the prevalence of ESA usage was lower in the NTG than in the CG at the same (or even higher) hemoglobin levels. All these favorable changes occur together with higher serum albumin levels in NTG patients. In the CKD 4 subgroup, the older age of the controls might contribute to low albumin levels. However, lower albumin levels in the control group were even more evident in CKD 5, where no difference of age exists.

The term “nutritional therapy” holds the potential to ensure that no patient is excluded and intervention is based on the individuation of the single patient’s nutritional needs. Nevertheless, dietary prescriptions are a rather infrequent practice in many parts of the world. In a recent survey in Italy, nutritional prescriptions were given to 54.8 % of patients with CKD stage 4 and to 65.9 % of patients with CKD stage 5 [7].

A restrictive or schematic approach, mainly based on eGFR level, may remain suitable in the younger cohort of CKD patients generally characterized by a nearly normal/high food intake, which likely induces metabolic abnormalities in the presence of moderate to severe renal insufficiency. In contrast, especially for elderly patients, it is quite prevalent to observe an under-nutrition pattern with only small increment of phosphate and urea serum levels: therefore special focus must be given to the energy intake in the elderly and/or frail patients.

A proper nutrition in CKD patients is able to correct or prevent signs, symptoms and complications of CKD, to delay the start of dialysis, and to prevent malnutrition. However, not all the patients have the same nutritional concerns and need the same intervention. The diet must be tailored to the single patient and the efficacy and safety of the diet is largely dependent on its feasibility.

Hence, based on the existing dietary habits, the dietary changes should be targeted to obtain specific goals (i.e. lowering urea production and/or lowering phosphate load, and/or modulation of sodium and energy intake, and so on…). During the follow up we adjusted the diet according to clinical, nutritional and biochemistry evaluation. In our experience with this approach patients’ adherence increases as their habits are not completely disrupted but gradually changed.

This approach particularly fits to the elderly patient who often have several barriers preventing correct feasibility of the dietary prescriptions such as socio-economic problems, chewing difficulties, scarce appetite, solitude or depression. In these cases, under-nutrition is quite prevalent so the priority is to increase food intake and energy intake, rather than restricting phosphate or protein intake, that is preventing malnutrition.

The NTG patients were given ND-LPD-VLPD or changes limited to energy, and/or sodium and/or phosphate when needed to correct metabolic or clinical abnormalities. As a whole, the distribution of the type of the nutritional therapy (as classified by protein content) is reported in Fig. 1.

We usually start from the existing dietary habits and we implement gradual changes to obtain the requested targets, using both written recommendations and visual tools that can be more impressive.

In practice, the first step consists of general advice to implement a healthy diet and to control salt and phosphate intakes avoiding high protein intake, in order to achieve normalization of protein and salt intake (0.8 g/Kg/day and 6 g/day respectively) according to the WHO recommendations for the general population [12, 13]. Low protein or very low protein regimens are prescribed as needed [3, 5] usually when residual renal function is critically reduced and overt metabolic abnormalities arise. It is noteworthy that, at the same protein intake, special attention has been paid to limit phosphate load as much as possible, especially to avoid processed food and products containing phosphate-based preservatives [20]. Energy prescription was dependent on requirement and protein intake. For those on protein restricted regimens, energy intake must equal or even overcome the energy requirement, including overweight or obese patients. Conversely, when low-energy intake regimens are necessary, protein intake must not be restricted as to avoid the risk of negative nitrogen balance [23].

Our findings are in keeping with previous reports which demonstrated favorable effects of renal diets [24–27]. The novelty of this paper is found in the implementation of a personalized, step-wise nutritional approach, that is tailored to the needs of the individual patient and designed to obtain specific nutritional targets.

Information coming from the Dietary Satisfaction Questionnaire was interesting . Patients reported a good rating of satisfaction with their diet, with a dislike rating reported by only 1 out of 10 patients. LPD patients showing a worse satisfaction than ND patients, likely because of more restricted protein intake and food choices due to the more severe residual renal function, and to the use of protein-free products. Patients on ND (0.8 g protein /Kg/day) were more satisfied with taste and flavor and the variety of the food eaten and stated they have no problems in finding food required for the diet with respect to 70 % of LPD (0.6 g protein/kg/d) patients. The majority of patients reported to have no difficulties in organizing their meals but LPD had more problems in eating out at restaurant or at someone’s home. Patients stated to be highly motivated to follow the diets with a higher percentage for LPD (90 vs 83.3 %) and to follow the diet at every meal (88.3 vs 69.7 %, *p* < 0.05): the more advanced CKD and the fear of dialysis commencing may account for these results. The low rating of “dislike” as regards the satisfaction of the eating pattern, 7.3 % for the ND patients and 13.1 % for the LPD patients, is encouraging as the success and safety of dietary treatment is related to the patients’ adherence and this is strictly related to their rating of satisfaction with the dietary patterns [10].

Finally, lower use of erythropoiesis stimulating agents, phosphate binders and active Vitamin D preparations was detected in NTG. Additional ad hoc studies are needed to confirm a favorable cost-effectiveness effect of the nutritional support [28].

The limitations of the study are mainly related to design and measurements.

The study is case-control and can not give the evidence of a randomized controlled trial. However, a case-control study allows interpretative evaluations of similar topics and of two different groups. Not all nephrology units are able to deliver nutritional interventions, but the activities of this clinic experience could be replicated.

It is possible that, in part at least, the NTG group looked better because the patients were more compliant or had a more receptive attitude to dietary intervention: however, nutritional support requires the active role of the patient to be effective and safe. The main reason why the CG patients did not receive any nutritional support was the lack of a renal dietician service, and the fact that physicians doubt the usefulness of the approach as well as the patient’s adherence. The two groups were very similar regarding eGFR levels and co-morbidities, and all patients were clinically stable and studied out of periods of acute illness.

The pharmaco-economic aspects were not addressed directly, but it is reasonable that nutritional support could reduce drug cost burden [28–30]. We hope that this study will stimulate further studies able to assess the cost-benefit of nutritional therapy.

## Conclusions

In summary, this case-control study shows the usefulness of a nutritional support in addition to the pharmacological good practice in CKD patients on tertiary care. At the same residual renal function, lower phosphate and BUN levels were obtained together with maintenance of serum albumin. In addition, a lower need of erythropoiesis stimulating agents, phosphate binders and active Vitamin D preparations was detected in NTG.

This study suggests that a nutritional support may be useful in the management of the world-wide growing CKD burden.

## Abbreviations

ACEi: Angiotensin-converting-enzyme inhibitor
ARB: Angiotensin II receptor blocker
BUN: Blood urea nitrogen
CG: Control group
CKD: Chronic kidney disease
DBP: Diastolic blood pressure
EAA: Essential amino acid
eGFR: estimated Glomerular Filtration Rate
ESA: Erythropoiesis stimulating agents
ESRD: End stage renal disease
HDH: Healthy dietary habits
KA: Keto-acids
LPD: Low protein diet
ND: Normal diet
NTG: Nutritional Therapy Group
PP: Pulse pressure
RDA: Recommended dietary allowance
SBP: Systolic blood pressure
VLPD: Very-low protein diet
